# LEGO: a novel method for gene set over-representation analysis by incorporating network-based gene weights

**DOI:** 10.1038/srep18871

**Published:** 2016-01-11

**Authors:** Xinran Dong, Yun Hao, Xiao Wang, Weidong Tian

**Affiliations:** 1 State Key Laboratory of Genetic Engineering, Department of Biostatistics and Computational Biology, School of Life Sciences, Fudan University, Shanghai 200436, P.R. China; 2 State Key Laboratory of Genetic Engineering, Collaborative Innovation Center of Genetics and Development, Department of Biostatistics and Computational Biology, School of Life Sciences, Fudan University, Shanghai 100433, P.R. China; 3 Children’s Hospital of Fudan University, Shanghai 200433, P.R. China

## Abstract

Pathway or gene set over-representation analysis (ORA) has become a routine task in functional genomics studies. However, currently widely used ORA tools employ statistical methods such as Fisher’s exact test that reduce a pathway into a list of genes, ignoring the constitutive functional non-equivalent roles of genes and the complex gene-gene interactions. Here, we develop a novel method named LEGO (functional Link Enrichment of Gene Ontology or gene sets) that takes into consideration these two types of information by incorporating network-based gene weights in ORA analysis. In three benchmarks, LEGO achieves better performance than Fisher and three other network-based methods. To further evaluate LEGO’s usefulness, we compare LEGO with five gene expression-based and three pathway topology-based methods using a benchmark of 34 disease gene expression datasets compiled by a recent publication, and show that LEGO is among the top-ranked methods in terms of both sensitivity and prioritization for detecting target KEGG pathways. In addition, we develop a cluster-and-filter approach to reduce the redundancy among the enriched gene sets, making the results more interpretable to biologists. Finally, we apply LEGO to two lists of autism genes, and identify relevant gene sets to autism that could not be found by Fisher.

Modern high-throughput functional genomics technologies are on one hand creating opportunities for exploring new biological frontiers, but on the other hand exhausting the scientific community’ resources to understand the information made available by such technologies. Biologists’ hardship in the face of this large-amount of experimental results calls for an automated tool to generate a high-level view of the inter-relations between existing biological knowledge and new experimental results. This is the place where Over-Representation Analysis (ORA) comes to rescue, helping to identify pathways or gene sets that are over-represented among a list of interesting genes produced from genomics studies, and thus provides biologists much needed computerized assistance in discovering novel genetic functional mechanisms.

A common solution for the ORA problem is to count the number of genes in the list of interesting genes that hit the gene set annotated by functional category such as Gene Ontology (GO)^1^, KEGG^2^, BioCarta^3^, REACTOME^4^, and MSigDB^5^, and then assess the significance of the overlaps using statistical methods such as Fisher’s exact test or chi-square test. Popular tools implementing this idea include DAVID^6^, BINGO^7^, GOEAST^8^, and GeneMania^9^,^10^, etc. Depending on the definition of a functional category, a gene set may refer to a collection of genes belonging to the same pathway or biological process, such as a KEGG pathway or a GO biological process term, or a collection of genes with same functional characteristics, such as a GO molecular function term or cellular component term. In this study, we focus on gene sets that are a collection of genes in a pathway or a biological process. For these gene sets, the traditional ORA tools have inherent limitations in that genes inside a pathway or a gene set are treated with equal importance, ignoring the constitutive functional non-equivalence of genes. For example, a gene may act as the regulator of a number of genes inside the same pathway, and the perturbation of this gene may have a larger impact on the pathway than the perturbation of its target genes do.

To address these limitations, a number of methods have been developed to incorporate pathway topology information, specifically the Directed Acyclic Graph (DAG) of KEGG pathways, and are therefore classified as Pathway Topology (PT) analysis. The first of PT methods was the impact factor (IF) analysis^11^, which introduced a term of perturbation factor to measure a gene’s contribution to the perturbation of a signaling pathway by taking into consideration not only its gene expression change, but also the type of interactions (e.g., induction or repression) it has with upstream genes and its position in a signaling pathway. The IF analysis has been included in ROntoTools^12^. Other PT methods include SPIA^13^, CePa^14^, and so on. A review of these PT methods can be found in^15^. However, the PT methods are not directly applicable for enrichment analysis of GO biological process terms or gene sets that are simple collection of genes in a pathway and lack any graph structure that explains the relationships between genes.

Recently, there are several network-based methods that deal with the ORA problem from a network perspective by incorporating gene-gene relationships. For example, NOA^16^ assigns GO terms to network links that connect two genes annotated with the same GO term, and measures the over-representation of GO terms links in a given network; NEA^17^ and EnrichNet^18^ both assess the over-representation of functional associations between a list of interesting genes and a given gene set in a functional association network. However, none of these methods take into consideration the functional non-equivalence roles of genes in a pathway. In addition, these methods are generally computational inefficient because they often employ network rewiring in order to evaluate the significance of the test statistics, and some of them are biased by the network structure.

Previously, we have developed a network-based method named GANPA for gene expression-based gene set enrichment analysis^19^. GANPA measures gene weights in a gene set according to its relative association with genes inside and outside the gene set in a functional association network, with higher weight indicating a more central role in the gene set. Then, gene weights are incorporated with expression change value to detect gene sets with significant expression change. GANPA and numerous methods with similar purpose, including GSEA^5^, PLAGE^20^,^21^, GLOBALTEST^22^, PADOG^23^, MRGSE^24^, GSA^25^, etc., are classified as the tools for Functional Category Score (FCS) analysis. The FCS problem is different from the ORA problem we are dealing with here: (1) FCS analysis takes the input of profiles of gene expression values, while ORA analysis deals with only the input of interesting genes; (2) a FCS method computes the test statistics based on the gene set, while an ORA method computes it based on the interesting genes. Therefore, GANPA cannot be directly applied for ORA analysis.

In this study, we adopt GANPA’s network-based gene-weighting approach with modification to weight genes, and develop a novel method named LEGO (functional Link Enrichment of Gene Ontology or gene sets) for ORA analysis. LEGO assigns gene set-specific weights to both genes within a gene set and the neighboring genes that have strong functional association with the gene set, by considering not only their relative network association with a gene set, but also their interactions with the genes inside the gene set. The test statistic of LEGO is the average gene set-specific weights of the interesting genes. The statistical significance of the test statistic is assessed by permutation in LEGO. As a network-based method, we first compare LEGO with Fisher and three other network-based methods—NOA, NEA, and EnrichNet using three benchmark datasets, and show that LEGO outperforms these methods in detecting functionally relevant gene sets. Recently, Tarca *et al*. compiled a benchmark of 42 disease gene expression datasets whose target pathways were known, and then compared 15 FCS methods and Fisher in terms of sensitivity, prioritization and specificity^26^. To further evaluate LEGO’s usefulness, from these datasets we select 34 datasets whose target pathways are KEGG pathways with DAG available, and compare LEGO with three PT methods—SPIA, ROntoTools and CePa, and five FCS methods—PLAGE, GLOBALTEST, PADOG, MRGSE and GSA that ranked top according to Tarca *et al*.^26^. The results show that LEGO is among the top ranked methods in terms of both sensitivity and prioritization for detecting target pathways, and its false positive rate is comparable to Fisher’s. In addition, we also develop a cluster-and-filter approach to reduce the redundancy among the enriched gene sets. Finally, we apply LEGO to two lists of autism-related genes identified by recent large-scale whole exome sequencing studies, and discover relevant gene sets to autism that could not be identified using Fisher. As such, LEGO will be of great value for identifying functionally relevant gene sets and deriving novel hypothesis in functional genomics studies.

## Materials and Methods

Given an interesting gene list *R* of size *n* and a gene set *S* of size *k*, the ORA problem is to test whether *S* is over-represented in *R*. Here, we introduce a network-based approach named LEGO. Below we describe the algorithm design of LEGO. The R package and instructions of LEGO can be downloaded at http://omics.fudan.edu.cn/static/softwares/Lego.tar.gz.

### LEGO’s algorithm design

#### Network-based gene set-specific weighting

Given a functional association network *G* with *m* genes, we adopt GANPA’s weighting approach with modifications to measure the gene set-specific weight for all genes. For details about GANPA, refer to^19^. Here, for every gene *i* in network *G*, we compute its degree inside the network as


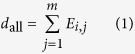


and its degree inside gene set *S* as


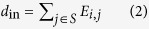


where *E*_*i,j*_ is the original edge weight for the functional link connecting gene *i* and *j* in network *G*. Under the assumption of equal probability for existing links between nodes, the expected degree of gene *i* to *S* is


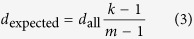


if gene *i* belong to *S*, or


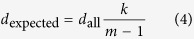


if gene *i* does not belong to *S*. A weighted score *W*_*i*_ that measures the relative network association of gene *i* with gene set *S* is then computed as:


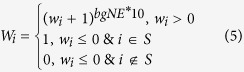


where


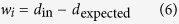


Both those genes within gene set *S* and those neighboring genes that have greater than expected association with gene set *S* are assigned a weight greater than 1 if *w*_*i*_ > 0. When *w*_*i*_ <= 0, if gene *i* belongs to S, then it is assigned a weight of 1; otherwise, it is assigned zero weight. In this formula, *bgNE* refers to the network efficiency^27^ of background network *G*, which is a measure for the density of a network. It is computed as


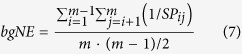


in which *SP*_*ij*_ is the shortest path between gene *i* and *j* in the network. The value of *bgNE* ranges from 0 to 1, with denser network having greater *bgNE* value. There are two reasons to use bgNE*10 here. (1) We want to assign more weights to genes that are more central to the pathway. (2) The variance of *w*_*i*_ in dense networks is much smaller than that in sparse networks because all nodes are highly connected to each other in dense networks; the use of bgNE*10 can compensate for this bias caused by the density of a network.

Having computed the gene set-specific weights for all genes in the network, we then assign gene set-specific edge weight for every functional link in the network as


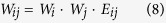


where *W*_*i*_ and *W*_*j*_ are the weights of gene *i* and *j*, respectively, and *E*_*i,j*_ is the original edge weight of the functional link. If there is no functional link for gene *i* and *j* in the network, then we define *W*_*i,j*_ = 0. For convenience, we also define 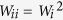. Finally, for every gene *i* in the network we calculate a gene set contribution score *G*_*i*_ by summing up the gene set-specific edge weights between gene *i* and all genes in *S* as:


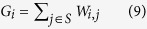


Thus, if a gene is more central to a gene set (i.e., with higher gene set-specific weight), and is functional linked to many “central” genes in the gene set, then it is assigned a higher gene set contribution score.

#### Test statistic and statistical inference

The test statistic of gene set *S* is simply the averaged gene set contribution score for all interesting genes in *R*, and is termed the Enrichment Score (*ES*_observed_). To evaluate the significance of the observed *ES*, we use the permutation strategy to generate the background distribution of *ES*: every time we randomly select the same *n* genes as the “interesting” genes, and compute the *ES*; this process is repeated 1000 times to obtain the null distribution for the *ES*, from which the p-value of the observed ES can be estimated. The use of permutation is convenient because we do not need to make any assumption for the real null distribution of *ES*. However, because of the limited number of permutations, the permutation approach often produces a p-value of zero. To avoid this problem, we follow the procedure proposed by Knijnenburg *et al*. to estimate the exact p-value by using the generalized Pareto distribution (GPD)^28^. Detailed procedures for p-value computation can be found in the supplementary materials.

### Multiple test correction

When there are more than one gene sets for analysis, we use bonferroni procedure to adjust the p-value^29^. We also report the adjusted p-value based on FDR^30^.

### Comparison of LEGO with Fisher and three network-based methods

Since LEGO is a network-based method for ORA analysis, we first compare LEGO with the traditional ORA method—Fisher’s exact test (named Fisher for convenience), and three recently developed network-based methods—NEA, EnrichNet, and NOA using three benchmark datasets. GeneMania^10^ is another network-based method for ORA analysis. But it is not compared here because its source code is not available upon request.

#### Descriptions of Fisher and three network-based methods

##### Fisher

For fair comparison with the network-based methods, genes in the background network are considered background genes, and are used to filter the gene set and the interesting gene list. A 2 × 2 contingency table is prepared for Fisher, with the rows corresponding to the number of genes inside the interesting gene list or not, and the columns corresponding to the number of genes annotated with the gene set or not. This table is then tested by *fisher.test*() in R.

##### NEA(version Dec 2012)^17^

NEA computes an Enrichment Score (*ES*_*observed*_) as the number of functional links between the interesting gene list and the gene set in the network. Then, it randomly rewires the network to re-calculate *ES*, in order to obtain the *Z*_*score*_ and the p-value of the *ES*_*observed*_. We download the R script of NEA from its website (http://www.meb. ki.se/~yudpaw/), and use seed 1234 suggested by the authors when rewiring the network.

##### EnrichNet (version 1.1)^18^

EnrichNet measures the functional association between the interesting gene list and a gene set using a Random Walk with Restart (RWR) algorithm. The RWR score of the gene set is compared to the RWR scores of all gene sets to derive an *Xd*_*score*_. According to^18^, *Xd*_*score*_ > *1.11* is approximate to p-value < 0.05, and is considered significant. We obtained the EnrichNet R Script directly from the authors. The web server of EnrichNet is http://www.enrichnet.org18.

##### NOA (version Apr 2012)^16^

The input of NOA is a network of interesting genes. NOA assigns a network link with a GO term if it connects two genes with the same GO term, and uses chi-square test to test the significance of GO term links in the input network as compared to that in a completely linked network based on the input genes. We use NOA’s webserver (http://app.aporc.org/NOA/) for enrichment analysis.

#### The three benchmark datasets

##### The yeast transcription factor (TF) dataset

A yeast TF co-regulatory network is downloaded from NOA^16^ (http://app.aporc.org/NOA/luscombe-trans-reg-net-all.id.TF.txt) and used as the background network (140 TFs, 2436 edges with no weight). Two lists of interesting genes (67 cell cycle related TFs (http://app.aporc.org/NOA/cellcycle.id.TF.txt) and 70 sporulation related TFs (http://app.aporc.org/NOA/sporulation.id.TF.txt)) and the GO terms are obtained from (http://app.aporc.org/NOA/). Here, the TF network is used as the background network.

##### The human breast cancer datasets

Three breast cancer gene expression datasets are downloaded from GEO database (GSE3744, GSE14548, GSE10780). R package *limma* is used to normalize the raw data. In each dataset, genes are ranked by their absolute t-statistics, and the top 200 differentially expressed (DE) genes are selected for enrichment analysis. The gene sets from MSigDB (version 4.0) (http://www.broadinstitute.org/gsea/msigdb/collections.jsp#C4)^5^ are tested. MSigDB is a collection of pathways from Biocarta, KEGG and Reactome. The human FunCoup functional association network (version 2.0) (http://funcoup2.sbc.su.se/download/Version_2.0/compact/FC2.hsapiens.compact.pfc01.tsv.gz) is used as the background network^31^ (17,083 genes, 4,353,383 edges with weight ranges from 0 to 1).

##### The human influenza host factors datasets

The human influenza datasets include four interesting gene lists that are obtained from^32^. These four lists of genes were originally obtained from four independent genome-wide RNAi studies that identified 237, 250, 287, and 294 host factor genes in response to influenza virus replication, respectively^33^,^34^,^35^,^36^. GO terms under the Biological Process branch are tested. The human GO term annotations (without IEA) are downloaded from http://cvsweb.geneontology.org/cgi-bin/cvsweb.cgi/go/gene-associations/gene_association.goa_human.gz (date: 11/25/2013)^37^. A total number of 3,445 GO terms with the size of 10 to 500 genes are selected for the test. The human FunCoup network is used as the background network.

#### Network edge removal and rewiring

For a given background network, to mimic network incompleteness and network noise we randomly remove or rewire a pre-defined percentage of edges in the network. Each time a different random seed is set. When rewiring the edges, we use the function *igraph_rewire_edges ()* from “igraph” package in R, which randomly rewires the endpoints of the edges uniformly to a new node with a constant percentage^38^.

#### Choice of different background networks

In the yeast TF dataset, another background network—YeastNet (354, 007 edges, with 5,702 genes) (http://www.inetbio.org/yeastnet/download.php?type=1)^39^ is also tested. In the human breast cancer datasets, we also select the STRING network^40^,^41^ (http://string-db.org/newstring_download/protein.links.v9.1/9606.protein.links.v9.1.txt.gz, 19,038 genes, 2,271,610 edges with weight ranges from 0 to 1) and HumanNet^42^ (http://www.functionalnet.org/humannet/HumanNet.v1.benchmark.txt) (4,086 nodes, 232,643 edges with no weight) as the background network, respectively. Then, LEGO is re-applied to calculate gene weights, and conduct gene set enrichment analysis as described above.

### Comparison of LEGO with Fisher, three PT methods and five FCS methods using 34 disease gene expression datasets

To further evaluate LEGO’s usefulness, we also compare LEGO with Fisher, three PT methods and five FCS methods using a benchmark of 34 disease gene expression dataset compiled by Tarca *et al*.^26^. In this benchmark, we use the STRING network as the background network.

#### The 34 disease gene expression datasets

Recently, Tarca *et al*.^26^ compiled 42 disease gene expression datasets (24 in the R package KEGGdzPathwaysGEO and 18 in the R package KEGGandMetacoreDzPathwaysGEO) whose target pathways were known (34 in KEGG and 8 in Metacore Disease Biomarker Networks). The gene sets to be tested are 259 KEGG pathways (R package KEGGESET) and 88 Metacore Disease Biomarker Networks (https://portal.genego.com (date stamp 4/19/2013)). Since KEGG pathways can be tested by the PT methods, we select the 34 datasets whose target pathways are KEGG pathways as the benchmark. We use the moderated t-test^43^ in R “limma”^44^ package to compute the p-value of differential expression for all genes. Then, we follow Tarca *et al*.’s procedures to identify DE genes : i) selecting more than 200 genes with FDR adjusted p-values<0.1; ii) if not, selecting more than 200 genes with original p-values <0.05 and log (fold change)>1.5; iii) if not, selecting top 1% of genes ranked by p-values.

#### Description of the three Pathway Topology-based (PT) methods

A recent paper reviewed 22 PT methods for enrichment analysis^15^. Two of them are actually network-based methods: one is GANPA from which LEGO is based, and another is EnrichNet that has been compared with LEGO in the three benchmark-datasets mentioned above. These two methods are not compared here. For the remaining 20 methods, 7 have available R scripts or packages for downloading; however, only ROntoTools^11^,^45^,^46^,^47^, SPIA^48^, and CePa^49^ can run without any additional packages. These three PT methods are compared with LEGO. Below we briefly describe each method.

##### ROntoTools

Pathway-Express in ROntoTools incorporates pathways topology to calculate a global probability for genes in a given pathway. The negative log value of this global probability is defined the impact factor of a gene, and the impact factor values are then used for pathway enrichment analysis^11^,^48^,^50^. This method is contained in the R package “ROntoTools” and the function “pe” was used.

*SPIA* is similar to ROntoTools except for the way to calculate the perturbation of a single gene. SPIA calculates the individual gene perturbation by subtracting the measured fold change, while ROnto-Tools calculates the gene perturbation as the sum of the fold change and the perturbation coming from upstream gene^48^. The “spia” function of “SPIA” package in R was performed.

*CePa* incorporates network centralities to weighted nodes from differentially expressed genes or gene expression data sets for pathway enrichment^49^. “cepa.all.parallel” of “CePa” package in R was used.

The input for ROntoTools, SPIA is a list of DE genes with fold change values. Cepa can take the input of either DE genes with fold change values or the matrix of gene expression profiles, and the corresponding methods are named CePa_dif and Cepa_mat, respectively.

#### Description of the five FCS methods

Tarca *et al*. developed measures of sensitivity (median p-value for the target pathways), prioritization (median rank for the target pathways), and false positive rate for comparing different methods. Here, we select the top five FCS methods ranked by Tarca *et al*. to compare with LEGO, which are PLAGE^20^,^21^, GLOBALTEST^22^, PADOG^22^, MRGSE^24^ and GSA^51^. The input of these five FCS methods is the matrix of gene expression profiles.

*GLOBALTEST* uses the empirical Bayesian generalized linear model to evaluate whether a gene set’s gene expression pattern is related to the phenotype^22^. The function “gtKEGG” of “globaltest” in R was used.

*GSA* uses the *maxmean* as the statistics to test whether the genes in a particular gene set have coordinated changes^51^. We used the function “GSA” of the GSA package in R.

*MRGSE* tests if the ranks in a particular gene set (sorted by the t-statistics) are different from genes in the background gene list^24^. We use moderated t-test in “limma” package in R to calculate t values and “geneSetTest” function in “limma” package for ranking of enriched gene sets.

*PADOG* gives higher weights for gene set specific genes and down weights the ubiquitous genes, and then integrates the t statistics and the gene weights to test the significance for each gene set^23^. The “padog” function of “padog” package in R was used.

*PLAGE*^20^,^21^ uses singular value decompositions (SVD) to reduce the dimensionality of gene expression variance for each gene set in each sample, defining as the “activity” score. Then, it tests the difference of this score between different phenotypic samples. We use the method “plage” in R package “GSVA”.

The above five FCS methods either use “sample permutation” or “gene permutation” strategy for statistical inference of the significance of gene set enrichment. Here, for all these methods, 1000 permutations are conducted.

### The measures of sensitivity, prioritization and false positive rate proposed by Tarca *et al*

Tarca *et al*.^26^ developed a number of measures for evaluating and comparing the performance of different methods. We also use these measures in the benchmark. Here, we briefly describe each of the measures. The sensitivity of a method is the median p-values of the target pathways in 34 datasets, with lower p-value indicates higher sensitivity. In each dataset, all tested pathways are sorted by their p-value (from lowest to highest), and the rank percentage of the target pathway is calculated. The prioritization of a method is the median values of the rank percentage of the target pathways in 34 datasets. For false positive rate (FPR), 50 times of random sample permutation are performed; then, the mean of the percentage of significant pathway (using a significance threshold of either 0.01 or 0.05) in all permutations is considered the FPR for each algorithm.

### A cluster-and-filter approach to reorganize the enriched gene sets

Because a gene can be annotated to different gene sets, there are many gene sets in gene set annotation database that are highly overlapping, especially for GO in which GO terms are organized in parent-child relationships. Since for most methods each gene set is tested independently, there may be significant redundancy among enriched gene sets, making it difficult for biologists to interpret the results. Here, we develop a cluster-and-filter approach to reduce the redundancy. It involves two steps: the clustering of all gene sets according to the degree of relatedness between gene sets before the enrichment analysis, and the filtering of enriched gene sets based on the clustering results. In the clustering step, given a gene set annotation system, such as MSigDB or GO database, we first construct a gene set-based network in which nodes correspond to gene sets, and edge weights represent the relatedness between two gene sets. The relatedness is defined by the Jaccard similarity (the number of overlapped genes/the number of union genes between two gene sets). To reduce the complexity of the network, here we only keep those edges whose similarity is greater than 0.15. Then, we partition the network into gene-set modules using iNP^52^, an iterated network partition algorithm. Finally, we obtain a number of gene-set modules each containing a list of gene sets that are overlapping, while the relatedness between gene sets from different modules is low. In the filtering step, given a list of enriched gene sets, we first group them according to the pre-determined gene-set modules they belong to. Then, we select the most significant gene set within each module as the marker gene set, and obtain the reduced enriched gene sets in which gene sets have low relatedness to each other. However, we also list the remaining enriched gene sets under their corresponding marker gene sets.

### Application of LEGO to the human autism datasets

The human autism dataset includes 22 and 27 autism-related genes obtained from Rubeis *et al*.^53^ and Iossifov *et al*.^54^, respectively, using large-scale exome sequencing. Here, we use human FunCoup network as LEGO’s background network, and MSigDB gene sets are tested.

## Results

### A brief description of LEGO

**Figure 1:**
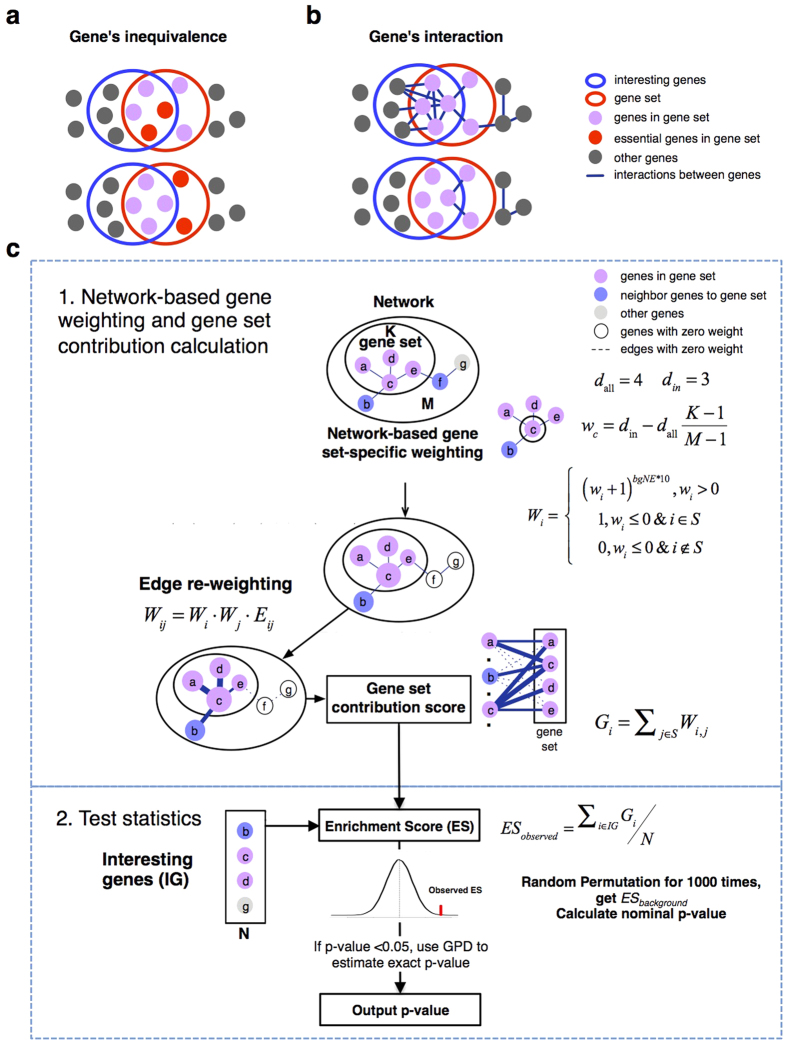
Brief description of LEGO’s algorithm design. (**a**,**b**) The limitations of traditional ORA method in distinguishing situations where all parameters are the same, but the overlapped genes are different in terms of gene’s inequivalence (**a**) or genes’ interactions (**b**). (**c**) The algorithm design of LEGO (see Methods for details). In the weighted network, gene weights are represented by node size. The weights of re-weighted edges are represented by the thickness of the edges.

Traditional ORA methods such as Fisher’s exact test (named Fisher for convenience) treat genes in a gene set or a pathway simply as gene labels with equal importance, and then test the significance of the over-representation of the gene set (the overlapped genes) among a list of interesting genes. Thus, they are unable to distinguish occasions where the overlapped genes are more essential to the gene set (Fig. 1a), or have more interactions between each other (Fig. 1b). To overcome these limitations, here we develop a novel method named LEGO (functional *L*ink *E*nrichment of *G*ene *O*ntology or gene sets). The workflow of LEGO is shown in Fig. 1c (For details about LEGO, refer to the Method section). Briefly speaking, we adopt a network-based gene weighting approach to assign gene set contribution scores to all genes in a functional association network. Then, we calculate the test statistic for a gene set as the mean gene set contribution score for the interesting genes, and use permutation to obtain the p-value of the test statistic. To avoid the zero p-value problem caused by limited number of permutations, we follow Knijnenburg *et al*. to use the generalized Pareto distribution (GPD) to estimate the exact p-value^28^.

### Comparison of LEGO with Fisher and three network-based methods in three benchmark datasets

Because LEGO is a network-based method for ORA (gene set Over-Representation Analysis) analysis, we first compare LEGO with Fisher, and three network-based methods—NEA, EnrichNet and NOA (refer to the Method section for details about these methods). Three benchmark datasets are used: the yeast transcription factor (TF) dataset, the human breast cancer datasets, and the human influenza host factors datasets. Because NOA cannot be applied to weighted networks, it is only tested in the yeast dataset.

#### The yeast TF dataset

When benchmarking NOA, Wang *et al*.^16^ constructed a yeast TF co-regulatory network that consisted of 2,436 co-regulatory relationships between 140 TFs. A co-regulatory relationship was established if two TFs share at least one target gene. The transcription regulatory relationships were obtained from Luscombe *et al*.^55^ who investigated the condition-specific yeast transcription regulatory networks. In that study, if a TF not only was highly expressed, but also had at least one target gene differentially expressed under a given condition, Luscombe *et al*. defined the TF active in that condition. They have defined 67 and 70 TFs that are active in the cell cycle and the sporulation conditions, respectively. Since GO:0022402 (“cell cycle process”) and GO:0043934 (“sporulation”) are the two most relevant GO terms to these two conditions, Wang *et al*. examined the ranks of these two GO terms among the enriched terms in the two lists of TFs, and assumed that a successful method would place the relevant GO terms to high ranks. Here, we follow the same strategy to compare different methods.

**Figure 2:**
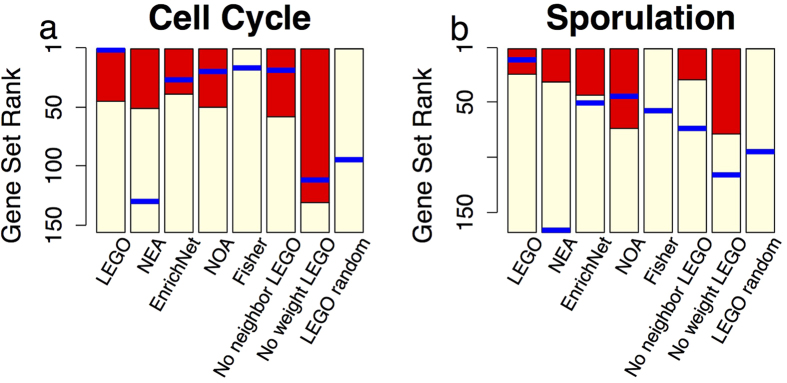
The ranks of the biologically relevant GO terms in the yeast dataset. GO term enrichment is conducted for 67 cell cycle-related (**a**) and 70 sporulation-related (**b**) yeast TFs by using different methods. Each bar represents the ordered GO terms based on the significance value produced by a method. The red box represents the significant GO terms (p-value < 0.05). The bold blue line corresponds to the location of the target GO term. LEGO random refers to the application of LEGO using randomly selected TFs. No weight LEGO refers to LEGO that assigns a weight of 1 to all genes of non-zero weights, and no neighbor LEGO refers to LEGO that assigns a weight of zero to all neighboring genes.

Among 183 GO terms that have more than five genes, 154 and 166 can be reported a significance value by all methods for the cell cycle and sporulation related TFs, respectively (Supplementary Table S1). Among all methods being compared, LEGO achieves the best ranks for the relevant GO terms in both the cell cycle and sporulation related TFs (Fig. 2). The cell cycle GO term is ranked 2^nd^ by LEGO, in contrast to 129, 27, 20, and 17 by NEA, EnrichNet, NOA, and Fisher, respectively (Fig. 2a). The sporulation GO term is ranked 11 by LEGO, in contrast to 165, 50, 44, and 57 by NEA, EnrichNet, NOA, and Fisher, respectively (Fig. 2b). However, if we randomly select 67 and 70 TFs for enrichment analysis and repeat the experiments 100 times, then the median ranks of the two relevant GO terms are about 90 by LEGO. The inclusion of both neighboring genes and appropriate gene weighting has a major impact on LEGO’s performance: if neighboring genes are excluded, the ranks of the cell cycle and sporulation GO terms would drop to 19, 73, respectively; if genes of non-zero weights are assigned a weight of 1, the ranks would drop to 111 and 115 (Fig. 2). To sum up, in this benchmark LEGO detects functionally relevant gene sets with the highest ranks among all methods.

#### The three breast cancer datasets

Large-scale functional genomics studies often involve the comparison of the enrichment results across independent datasets under similar studying condition, and it is expected that a successful method should produce consistent results. Here we select three independent breast cancer datasets, and perform enrichment analysis for the top 200 differentially expressed genes in each dataset. Then, we count the number of overlapped gene sets that are significant in at least two datasets at a given rank threshold (e.g., top 10, 20, …, 80 significant gene sets). The background network is the human FunCoup network^31^. Gene sets from MSigDB^5^ are tested. Multiple test correction is not implemented because the *Xd*_*score*_ of EnrichNet cannot be adjusted, while Fishers find only few significant gene sets after adjustment. The detailed lists of enriched gene sets identified by different methods can be found in Supplementary Table S2.

**Figure 3:**
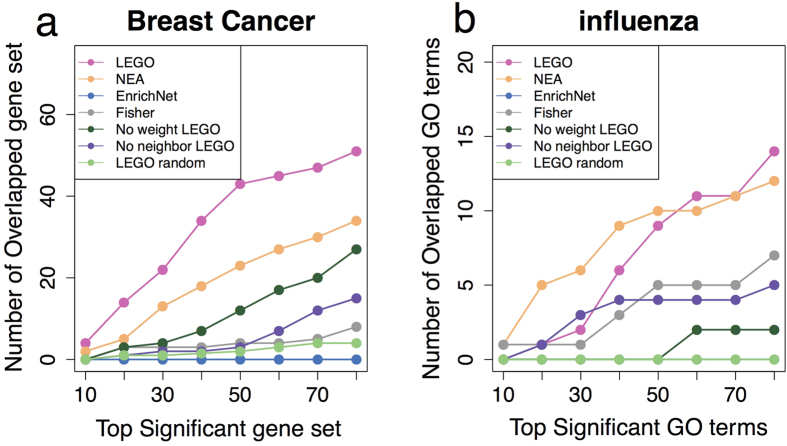
The overlaps among enriched gene sets identified from independent datasets under similar studying conditions. (**a**) Scatter plots of the number of overlapped significant MSigDB gene sets identified in three breast cancer datasets at different rank threshold. (**b**) Scatter plots of the number of overlapped significant GO terms under Biological Process branch identified in four influenza datasets at different rank threshold. No weight, no neighbor LEGO and LEGO random are the same as in Fig. 2.

In this experiment, LEGO identifies more overlapped gene sets than any of the methods being compared does at all rank thresholds (Fig. 3a). Of the top 10 significant gene sets found in each of the three breast cancer datasets, LEGO identifies four overlaps, which are “REACTOME_CYCLIN_A_B1_ASSOCIATED_EVENTS_DURING_G2_M_TRANSITION”, “REACTOME_E2F_MEDIATED_REGULATION_OF_DNA_REPLICATION”, “REACTOME_UNWINDING_OF_DNA” and “REACTOME_KINESINS”. One of the most important features of cancer cell is abnormal cell division in which normal cell cycle-related processes are significantly altered^56^. Here, these four gene sets all have to do with cell cycle, suggesting that cell cycle-related processes are significantly altered in breast cancer cells. From the top 80 significant gene sets, LEGO identifies 51 overlaps, significantly more than that found by the other methods. For majority of those overlapped gene sets, the abnormal functioning of their corresponding biological processes are relevant to cancer development, including “REACTOME_G1_S_SPECIFIC_TRANSCRIPTION”, “REACTOME_CELL_CYCLE_MITOTIC”, etc. Of the remaining methods, NEA identifies more overlapped gene sets than the other methods. However, NEA’s results are actually caused by the bias in its method (shown in later section).

The performance of LEGO is attributed to both gene weighting and the inclusion of neighbor genes: once gene weights or neighbor genes are removed, we observe a significant drop in the number of overlapped gene sets, especially for the removal of neighboring genes (Fig. 3a). Moreover, when 200 genes are randomly selected, we only observe a median of 4.0 overlapped gene sets among top 80 significant pathways by LEGO, suggesting that LEGO’s performance is not due to over-detection, i.e., a gene set is reported significant regardless of the input genes.

#### The four influenza host factors datasets

With similar purpose, here we examine whether LEGO can produce consistent enrichment results across four lists of human host factor genes in response to influenza virus replication that were identified by independent investigators^33^,^34^,^35^,^36^. The gene sets to be tested are GO terms under Biological Process branch. A GO term is considered “overlapped” if it is found significant in at least three of the four datasets. Again, LEGO identifies significantly more overlapped GO terms than Fisher and EnrichNet (Fig. 3b). EnrichNet could hardly get any significant enriched GO terms. After the threshold of top 60 GO terms, NEA detects more overlapped GO terms than LEGO. This is, however, because NEA is strongly biased and produces many GO terms with zero p-value. Nearly all GO terms found by Fisher are identified by LEGO. The unique GO terms identified by LEGO are relevant to the studying condition. For example, both “GO:0070972: protein localization to endoplasmic reticulum” and “GO:0045047: protein targeting to ER” are identified by LEGO but not by Fisher, and it has been suggested that protein localization and targeting to ER may play critical roles in the process of influenza virus replication^57^. As a comparison, when genes are randomly selected as host factor genes, LEGO does not identify any overlapped GO terms, while removal of gene weights or neighboring genes would result in the drops of overlapped GO terms (Fig. 3b). Thus, we conclude that compared to the methods being performed here, LEGO can detect more consistent biologically relevant gene sets across independent datasets under similar studying conditions.

### Investigation of network bias on LEGO

LEGO, NEA and EnrichNet are all network-based ORA methods. To investigate whether the topology of the background network or the gene set may cause some bias to the performance of these methods, we randomly select the same number of genes as that of the original interesting genes in both the yeast and human breast cancer benchmark datasets (e.g., in the yeast dataset, 67 TFs are randomly selected to mimic the 67 cell cycle-related TFs), and redo the ORA analysis. This procedure is repeated 100 times. Then, for each gene set we compute the frequency that it is found significant (p-value < 0.05 or *Xd*_*score*_>1.11 for EnrichNet) (termed frequency of random significance). Given the significance threshold is set at 0.05, the expected frequency of random frequency for a gene set should be 5%. However, since the experiments are only repeated 100 times, there may be random variations for some gene sets. But the median frequency should be at 5%, and the random variations would be reduced with more experiments conducted (Supplementary Fig. S1). The influenza benchmark is not re-analyzed because it is similar to the human breast cancer benchmark.

**Figure 4:**
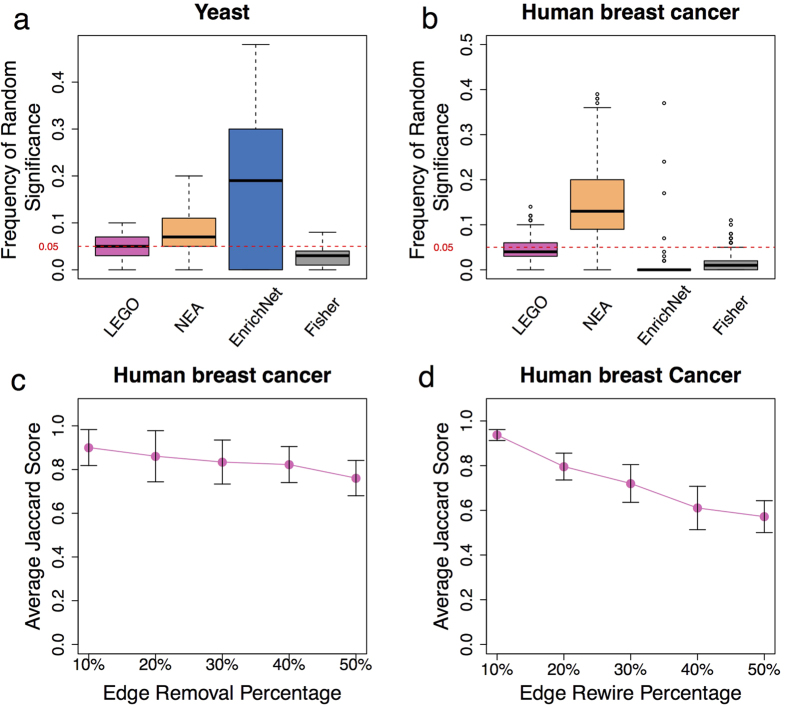
The impact of network bias and network perturbation on the performance of different methods. For network bias, (**a**,**b**) show the boxplots of the frequency of random significance (percentage of times a gene set is found significant (p-value < 0.05) with randomly selected genes) for 153 GO terms in the yeast dataset, and 1,077 MSigDB gene sets in the human dataset, respectively. For network perturbation, each time a portion of edges in the FunCoup network are randomly removed (**c**) or rewired (**d**), and LEGO is re-applied to the human dataset. The overlapped significant pathways (out of top 80) based on the perturbed network are compared to the results based on the unperturbed network, and a Jaccard similarity is computed (the intersection between the two lists of overlapped significant gene sets/the number of the original overlapped significant gene sets). Experiments are repeated 100 times, and the average and standard deviation are plotted.

In both the yeast and human breast cancer datasets, the median frequency of random significance for a gene set is lower than or approximately 5% by LEGO (Fig. 4a,b), indicating that there is no systematic bias in LEGO that makes some gene sets easily detected significant regardless of the input genes. Fisher is more conservative than LEGO, with the median frequency of random significance lower than 5% in both datasets. In contrast, both EnrichNet and NEA are strongly biased. EnrichNet tends to identify most gene sets with a frequency of random significance greater than 5% in the yeast dataset, while finds nearly no gene sets significant in human datasets (Fig. 4b). Since the density of these two networks is very different (the network efficiency (a measure of the density of a network) of the yeast, human networks is 0.61, 0.27 respectively), it is likely that EnrichNet is biased by the density of a network. For NEA, the median frequency of random significance is 17% in human dataset, indicating it tends to identify some gene sets as significant regardless of the input genes. In fact, in NEA the frequency of random significance is strongly positively correlated to both the network efficiency and the size of gene sets; in contrast, we find no such correlation in LEGO (Supplementary Fig. S2).

### The impact of network incompleteness, network noise and network choice on LEGO’s performance

Currently available functional association networks are not complete and may be noisy^58^. To inspect how this may affect LEGO’s performance, we mimic network incompleteness and network noise by randomly removing or rewiring a portion (e.g., 10%, 20%, …, 50%) of edges in the background network, and redo the analysis in the yeast and human benchmarks. Here, edge removal or rewiring would not remove genes from the background network, but would cause changes in the gene-gene interactions in the background network. As such, we have to re-calculate the gene set-specific gene set contribution scores for every gene in the background network after edge removal or rewiring, and then apply LEGO for enrichment analysis. In the yeast benchmark, we record the ranks of the two relevant GO terms; in the human benchmark, we identify the overlapped significant gene sets (top 80 significant gene sets), and compare them with the original results. At each portion of edge removal or rewiring, we repeat the experiments 100 times, and compute the average and standard deviation.

The yeast TF network is a small network, and network perturbations on this network have significant impacts on LEGO’s performance: both the cell cycle and the sporulation GO term are barely significant after 10% edge removal or rewiring, and their average ranks drops with the increase of network perturbations (Supplementary Fig. S3). In contrast, the FunCoup network is a much larger network, and LEGO is more resistant to network perturbations on this network. With the perturbed FunCoup network, LEGO still identifies significantly more overlapped gene sets than Fisher, and the overlapped gene sets are similar to those identified using the unperturbed network, although the similarity drops with more factions of the networks perturbed (Fig. 4c,d). Edge rewiring on the FunCoup network has a more significant effect on LEGO than edge removal does, which is because most genes have only a few interactions in the FunCoup network, making gene weighting more sensitive to edge rewiring. Thus, for large networks LEGO is generally resistant to network incompleteness and network noise, though its performance can be further enhanced with the addition of new interactions and the improvement in network quality.

We also inspect the choice of different types of networks on the performance of LEGO. In the yeast benchmark, we change the background network to YeastNet, a larger and high quality functional association network. YeastNet is very different from the yeast TF network in that: it consists of many more genes that are mostly non-TFs, and the interactions between genes are significantly different from that in the TF network. Consequently, both GO terms are not found significant using YeastNet. In the human breast cancer benchmark, we repeat the analysis using the human STRING network and HumanNet. Both FunCoup and STRING are large networks, and the gene weights calculated based on these two networks are positively correlated for majority of gene sets (Supplementary Fig. S4). In contrast, HumanNet is a probabilistic functional interaction network, and has much smaller gene coverage than the other two networks (for most gene sets, only less than half of the genes are covered in HumanNet). The results of LEGO using the STRING network are similar to that of using the FunCoup network, while they are worse when using HumanNet (Supplementary Fig. S5). Thus, for general purpose we recommend the use of large networks such as the FunCoup or the STRING network because a pathway would be better covered in large functional networks. However, when interesting genes are prepared for specific purpose or share some common properties, such as the active TFs in the yeast dataset, it may be necessary for users to provide their own background network, such as the TF co-regulatory network.

### Computational efficiency of LEGO

During the benchmark of LEGO, we find the other network-based methods are all computationally costive, especially for NEA. NEA takes 49 hours to complete the breast cancer benchmark and 170 hours for the influenza benchmark (Supplementary Table S3). This is mainly because these methods need to spend a large amount of time on rewiring a network, which is especially time consuming for large networks. In contrast, the major computation cost in LEGO is the network-based weighting process, particularly the calculation of background network efficiency, which can be done in advance. This leaves the major computational cost for LEGO to permutation. Therefore, LEGO can be very efficient in functional genomics studies.

### Comparison of LEGO with Fisher, three PT methods and five FCS methods using a benchmark of 34 disease gene expression datasets

In 2013, Tarca *et al*.^26^ compiled 42 disease gene expression datasets in which the target pathway of each dataset was known. They used these datasets to compare 15 FCS methods and Fisher. The target pathways of 34 out of the 42 datasets are KEGG pathways that have representative DAGs, which can be tested by PT methods. Here, to further test LEGO’s usefulness in functional genomics studies, we select the 34 disease gene expression datasets as the benchmark, and compare LEGO with Fisher, three PT methods—SPIA, CePa and ROntoTools, and the top five FCS methods ranked by Tarca *et al*.—PLAGE, GLOBALTEST, PADOG, MRGSE, and GSA. The descriptions of these methods can be found in Methods. We follow Tarca *et al*.’s procedures to identify the DE genes in each of the 34 datasets. LEGO and Fisher use the DE genes for pathway enrichment. SPIA, ROntoTools use DE genes with fold change values as input. CePa can use either DE genes or gene expression profiles for pathway enrichment, and we test both options for CePa and for convenience name them as CePa_dif and CePa_mat, respectively. All the other methods use matrix of gene expression profiles as the input, and we follow the instructions of their respective R package to perform pathway enrichment.

**Figure 5:**
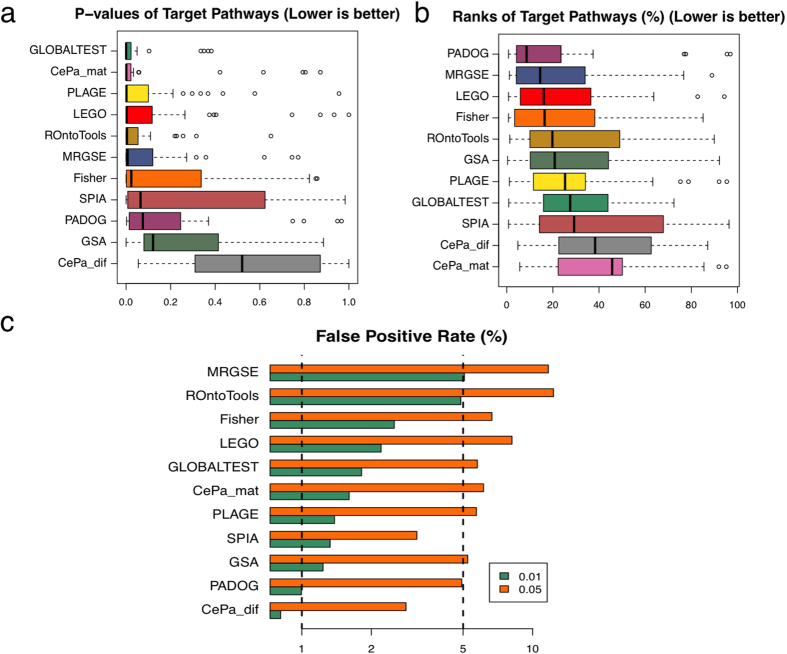
Comparison of the performance of different methods using a benchmark of 34 disease gene expression datasets. The p-values (**a**) and the ranks (%) (**b**) of the target pathways produced by different method are shown in boxplots. (**c**) The false positive rate of different methods is shown as the percentage of all pathways found significant at different significant level (0.01 and 0.05) from 50 sample permutation results. The vertical lines indicate the expected level of false positive rate at a given significance level.

Tarca *et al*. proposed three measures to evaluate the performance of a method, which are sensitivity (defined as the p-value for the target pathway), prioritization (defined as the rank of the target pathway), and false positive rate (see Methods for details). Here, we use the same measures to compare these methods. All results can be found in Supplementary Table S4. Out of the 11 methods (two options for CePa) being compared, LEGO ranks 4^th^ in terms of sensitivity (the median p-value for the target pathways) (Fig. 5a), and 3^rd^ in terms of prioritization (the median rank for the target pathways) (Fig. 5b). LEGO’s false positive rate is comparable to Fisher’s (Fig. 5c). It is worth noting that the sensitivity measure and the prioritization measure evaluate the performance of a method from different perspectives, and a method that ranks the best based on one measure may not also rank the best based on another measure. For examples, GLOBALTEST ranks 1^st^ in terms of sensitivity, while it ranks only 8^th^ in terms of prioritization; PADOG ranks 1^st^ in terms of prioritization, but it ranks only 9^th^ in terms of sensitivity. The fact that LEGO ranks among the top methods by both sensitivity and prioritization measures suggests that it is able to prioritize target pathways with high sensitivity. It is also worth noting that most of the methods being compared here use gene expression values, which contains more information than DE genes do. In conclusion, LEGO’s performance in this benchmark well illustrates its usefulness for functional genomics studies.

### A cluster-and-filter approach to reduce the redundancy among enriched gene sets

**Figure 6:**
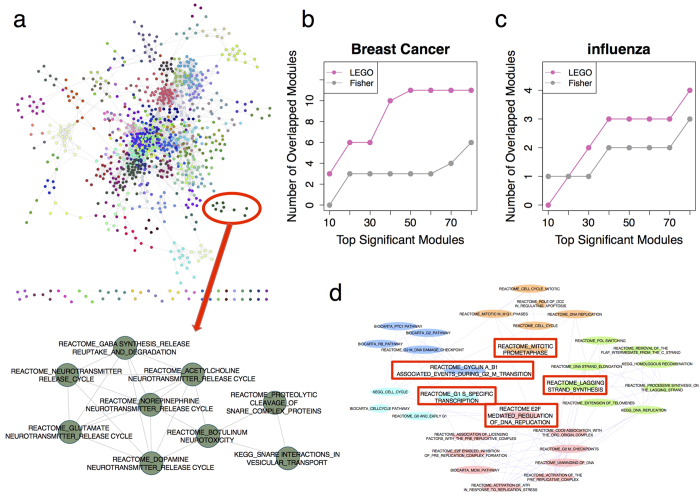
The cluster-and-filter approach to reduce the redundancy among enriched gene sets. (**a**) A network of 1,077 gene sets from MSigDB is presented, with nodes corresponding to gene sets and edge weights representing the relatedness between gene sets. This network is partitioned into modules of gene sets, with gene sets belonging to the same module marked with the same color. An example of a gene set module is shown at the bottom of the network. (**b**,**c**) are similar to Fig. 3a,b except that the overlapped significant MSigDB gene sets or GO terms are grouped according to the modules they belong to, and then the number of modules is plotted. (**d**) An example to show five selected gene-set modules based on the enriched gene sets identified by LEGO in a breast cancer gene expression dataset (GSE10780). Links between gene sets indicate they are related. Gene sets belonging to the same module are marked with the same color. The size of each node (gene set) is proportional to its significance, and the most significant gene set in each module is highlighted by the red-bordered box (the marker gene set).

Most ORA methods including LEGO perform enrichment test independently for each gene set, and may result in enriched gene sets that are highly overlapping, making it inconvenient for biologists to interpret the results. This is especially a problem for the enrichment of GO terms, as GO terms are organized in parent-child relationships. To reduce the redundancy among the enriched gene sets, here we develop a simple cluster-and-filter approach. The idea is that we cluster all gene sets into gene-set modules according to their degree of relatedness; then, given the enriched gene sets, we reorganize them according to the modules they belong to, and select the most significant ones from each module as the marker gene sets to display the results. Based on this idea, we have obtained 145 gene sets modules from 1,077 gene sets in MSigDB, and 1,410 GO modules from 12,062 GO terms under Biological Process branch (for details refer to Method section). In Fig. 6a, we show the global gene set network of MSigDB in which nodes correspond to gene sets, and edge weights represent the relatedness between gene sets. An example of a gene-set module is provided. This module includes nine gene sets that are all related to neurotransmitter release cycle that are highly overlapping. Clustering them into one module can thus help reduce the redundancy among enriched gene sets.

Originally, in the breast cancer benchmark, LEGO identifies 51 overlapped gene sets among top 80 significant gene sets. Using the cluster-and-filter approach, these gene sets are reduced to 12 modules, and the original 9 overlapped gene sets found by Fisher are now reduced to 6 modules (Fig. 6b). Similarly, the enriched GO terms in the human influenza benchmarks can also be reduced into GO term modules (Fig. 6c). For details about the reorganization of the enriched gene sets in these two benchmarks, refer to Supplementary Table S2. Here, we show an example of the reorganization of enriched gene sets in one breast cancer dataset (GSE10780). In this dataset, LEGO identifies 51 significant gene sets (p-value < 0.01 after FDR adjustment), which are reduced to 15 modules in which five are related to cell cycle. Inspection of these five modules reveals that they actually represent different stages of cell cycle (Fig. 6d). The gene sets within each module are highly overlapping, while those from different modules do not have significant overlaps. Thus, the reorganization of the enriched gene sets makes it easier for biologists to understand the enrichment results.

### Application of LEGO to two large-scale autism exome sequencing studies

**Figure 7:**
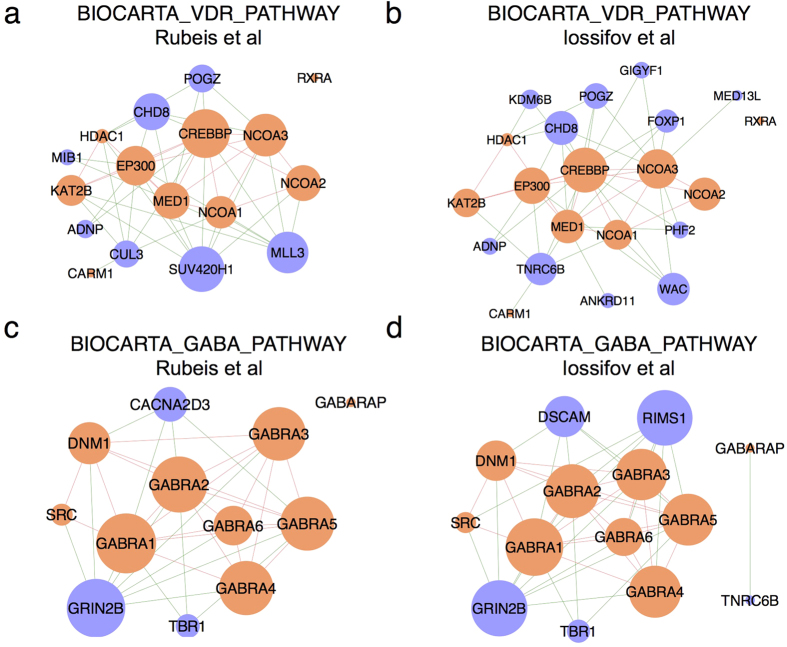
Autism-related genes are strongly associated with the two pathways identified by LEGO. The sub networks of FunCoup are shown. These sub networks consist of genes from “BIOCARTA_VDR_PATHWAY” (**a**,**b**) or “BIOCARTA_GABA_PATHWAY” (**c**,**d**), and the autism-related genes identified by Rubeis *et al*. (**a**,**c**) or Iossifov *et al*. (**b**,**d**) that are neighboring genes to these two pathways in the FunCoup network. Genes annotated to these two pathways are colored in orange, and the autism-related genes are colored in purple. Edges colored in red represent the links between genes inside the pathways, while those colored in green represent the links between the autism-related genes and the genes inside the pathways. The size of each node (gene) is proportional to the gene set-specific gene set contribution score calculated by LEGO.

After extensive benchmarks, we have demonstrated LEGO’s usefulness in functional genomics studies. Here, we apply LEGO to two lists of autism-related genes. Autism is a neurodevelopment genetic disease, and is characterized by social and communication disorder. Recently, Rubeis *et al*. and Iossifov *et al*. have both independently conducted large-scale exome sequencing on autism patients and normal people, and have identified 22 and 27 autism-related genes, respectively. Fisher’s exact test on these two lists of genes does do not find any significant MSigDB gene sets (p-value < 0.05 after FDR adjustment). In contrast, with LEGO we find 10 and 35 enriched MSigDB gene sets from the 22 and 27 autism-related genes, respectively (p-value < 0.05 after FDR adjustment), in which 7 gene sets are common (Supplementary Table S5). These enriched gene sets are further reduced to 6 and 13 gene sets modules, respectively, and the marker gene sets (the most significant gene set within a gene-set module) of two modules are common (Table 1). These two gene sets are “BIOCARTA_VDR_PATHWAY” and “BIOCARTA_GABA_PATHWAY”. Interestingly, there are no autism genes annotated to these two gene sets. The reason why LEGO finds these two gene sets significant is because the two lists of autism-related genes contain neighboring genes that have significant interactions with the genes inside these two pathways in the FunCoup Network (Fig. 7). We conduct a literature search, and find strong evidence that these two pathways are relevant to autism. VDR stands for Vitamin D receptor, and it has been reported that lower vitamin D levels in developing brains can increase the risk of autism disorder^59^. GABA (Gamma-amino butyric acid) is an inhibitor neurotransmitter, and acts as synapses by binding to GABA receptors. The GABA pathway is responsible for synaptic inhibition in brain, and has been strongly suggested to play a role in the etiology of autism disorder^60^. Therefore, it is likely that mutations in candidate autism genes (particularly those neighbor genes to these two pathways) may disrupt the normal functioning of these two pathways, which may then lead to the development of autism. In the original paper, because the 22 genes are a small list, Rubeis *et al*. relaxed the FDR criterion to collect 107 genes to perform gene set enrichment, and identified four enriched functional clusters including synaptic formation, transcriptional regulation and chromatin-remodeling pathways, cell junction and neurodegeneration. However, there are no those pathways in MSigDB. We then apply LEGO to test the enrichment of GO terms, and find some of the enriched GO terms are similar to the above four functions (Supplementary Table S5). Thus, besides the functions identified in the original study, LEGO is able to identify relevant functions to autism, helping to explain the molecular mechanism for the causal roles of some autism genes. This example further demonstrates LEGO’s usefulness in large-scale functional genomics studies.

## Discussion

The major limitations of traditional ORA methods, particularly the most widely used Fisher’s exact test, are that they ignore the functional non-equivalence roles of genes and the complex interactions between genes. In this study, we have developed a novel method named LEGO that takes a network-based approach to transform the functional non-equivalence roles of genes and gene-gene interactions into gene weights, and then incorporate the weights in ORA analysis. To test LEGO’s performance, we compare it with Fisher and three recently developed network-based methods using three benchmarks. In the yeast benchmark, we show that LEGO is able to identify the two GO terms—“cell cycle” and “sporulation” relevant to the cell-cycle-related and sporulation-related TFs, respectively, with higher ranks than the other methods do. In both the breast cancer and human influenza benchmarks, we show that LEGO detects more consistent gene sets across independent datasets under similar studying conditions than the other methods do, and also show those consistent gene sets are relevant to the studying condition. As an application of LEGO, we further apply it to two lists of autism-related genes, and show that LEGO can discover pathway relevant to autism that could not be found by Fisher. These lines of evidence well demonstrate LEGO’s advantage over traditional ORA methods such as Fisher for functional genomics studies.

The network-based gene weighting is a critical step in LEGO. Below, we give a few examples from both yeast and human datasets to show that LEGO’s gene weights can truly reflect the functional non-equivalence roles of genes within a pathway. In yeast, RIM101 has the highest weight in the GO term “Sporulation”, while OAF1 has the lowest weight. RIM101 is required for alkaline pH-stimulated haploid invasive growth and sporulation^61^, while OAF1 is an oleate-activated transcription factor involved in fatty acid biogenesis^62^, and is actually ranked top 1 in “GO:0044255 cellular lipid metabolic process”. In human, TP53 is the central gene in both p53 pathway^63^ and p53 hypoxia pathway^64^, and is ranked top 2 and top 3 in both pathways. Meanwhile, TP53 is ranked only 108 for the pathway of Huntington’s disease (Supplementary Table S6). EGFR is ranked top 1 in EGF SMRTE pathway.

With the use of network in LEGO, locally the topology, coverage and size of a pathway in the network, and globally the network incompleteness, network noise, and the type of network interactions can all potentially affect the calculated gene weights, and consequently the enrichment results of LEGO. We have rigorously investigated those effects on LEGO’s performance by using randomly selected interesting genes, randomly removing or rewiring network edges, and comparing the use of different types of networks. We have demonstrated that there is no systematic bias in LEGO that makes some pathways easily detected as significant regardless of the input genes. However, the use of a different network may result in the change of gene set-specific weights in LEGO, leading to different results for some gene sets. In general, when a network covers more genes with interactions of higher quality and a gene set is better represented in the network, LEGO will be more useful. On the other hand, since different networks may have different coverage for a given pathway, in practice it may be useful to conduct enrichment analysis with several available networks, or users can provide their specially designed networks. Furthermore, when a network is updated with more interactions or the corpus of gene annotations increases, LEGO’s pathway-specific gene weights will have to be recalculated.

Besides comparing LEGO with Fisher and three network-based methods, in this study we also compare it with three Pathway-Topology (PT)-based methods and five Functional Category Score (FCS) methods using a benchmark of 34 disease gene expression datasets prepared by Tarca *et al*.^26^, and show that it is among the top ranked methods in terms of both sensitivity and prioritization for detecting target pathways. Both LEGO and the PT methods assign weights to genes before calculating the test statistics of a given gene set. The differences between them are the followings. (1) The PT methods use the pathway topology, specifically the DAG representation of a pathway, to infer weights to genes inside a pathway. In contrast, LEGO uses network topology to infer gene weights, and does not require the input of the DAG representation of a pathway. (2) The PT methods do not use gene-gene interactions between genes inside a pathway and those outside the pathway, whereas LEGO considers not only genes inside a pathway, but also the neighboring genes that have strong association with the pathway. In fact, the inclusion of neighboring genes plays an important role in LEGO’s performance, which is well illustrated in the three benchmarks comparing LEGO with other network-based methods and in the autism dataset. FCS methods incorporate gene expression values when assessing the significance of a pathway, whereas LEGO takes only the input of differentially expressed (DE) genes. Although LEGO performs fairly well compared with the five FCS methods being compared in this study, it is expected that the incorporation of gene expression values in LEGO may further improve its performance. We are now working to extend LEGO for FCS analysis by incorporating gene expression change values.

The redundancy of enriched gene sets is a common problem for most methods including LEGO, because they test the significant of a gene set independently and many gene sets, especially GO terms, are highly overlapping with each other. Recently, multivariate model-based gene set analyses, such as MGSA^65^ have been proposed to solve this problem. MGSA takes into consideration the specificity of different gene sets when evaluating their significance, and automatically produce enriched gene sets that have low redundancy. However, MGSA does not find any significant gene sets in either the human breast cancer or the human influenza dataset according to significance threshold suggested by the authors (Supplementary Table S2), indicating that the efficiency of these methods needs to be improved. In this study, we develop a simple cluster-and-filter approach to reduce the enriched gene sets into gene-set modules that have low redundancy. With the reduced gene-set modules, the enrichment results can be organized in a more friendly way for biologists to interpret. In the future, it may be needed to combine the cluster-and-filter approach with the multivariate module-based analysis to further refine the enrichment results. Nevertheless, the benchmark results of LEGO and the adoption of the cluster-and-filter approach to post-process LEGO’s result already make LEGO a useful method for large-scale functional genomics studies.

## Additional Information

**How to cite this article**: Dong, X. *et al*. LEGO: a novel method for gene set over-representation analysis by incorporating network-based gene weights. *Sci. Rep*. **6**, 18871; doi: 10.1038/srep18871 (2016).

## Supplementary Material

Supplementary Information

Supplementary Table S1

Supplementary Table S2

Supplementary Table S3

Supplementary Table S4

Supplementary Table S5

## Figures and Tables

**Table 1:** The enriched gene set modules found by LEGO among the autism genes that are consistent in between the studies of Rubeis *et al*. and Iossifov *et al*.

	Enriched pathways from the 22 autism genes identified by Rubeis *et al*.	Enriched pathways from the 27 autism genes identified by Iossifov *et al*.
Gene set module 1	Marker pathway of the module BIOCARTA_VDR_PATHWAY (7.0e-4);	Marker pathway of the module
	Remaining pathways belonging to the module	BIOCARTA_VDR_PATHWAY (0.038);
	REACTOME_YAP1_AND_WWTR1_TAZ_STIMULATED_GENE_EXPRESSION (7.0e-4);	Remaining pathways belonging to the module
	REACTOME_BMAL1_CLOCK_NPAS2_ACTIVATES_CIRCADIAN_EXPRESSION (2.2e-3);	REACTOME_CIRCADIAN_REPRESSION_OF_EXPRESSION_BY_REV_ERBA (0.038);
	REACTOME_CIRCADIAN_REPRESSION_OF_EXPRESSION_BY_REV_ERBA (2.5e-3);	REACTOME_YAP1_AND_WWTR1_TAZ_STIMULATED_GENE_EXPRESSION (0.042);
	REACTOME_CIRCADIAN_CLOCK_0.0028111;	
	REACTOME_RORA_ACTIVATES_CIRCADIAN_EXPRESSION (3.5e-3);	
Gene set module 2	Marker pathway of the module BIOCARTA_GABA_PATHWA (0.03);	Marker pathway of the module
		BIOCARTA_GABA_PATHWAY (4.8e-3);
		Remaining pathways belonging to the module
		REACTOME_GABA_A_RECEPTOR_ACTIVATION (0.038)

## References

[b1] Consortium, G. O. Gene Ontology annotations and resources. Nucleic acids research 41, D530–D535 (2013). 23161678 10.1093/nar/gks1050 PMC3531070

[b2] Kanehisa M., Goto S., Furumichi M., Tanabe M. & Hirakawa M. KEGG for representation and analysis of molecular networks involving diseases and drugs. Nucleic acids research 38, D355–D360 (2010). 19880382 10.1093/nar/gkp896 PMC2808910

[b3] Nishimura D. BioCarta. Biotech Software & Internet Report: The Computer Software Journal for Scient 2, 117–120 (2001).

[b4] Croft D. . The Reactome pathway knowledgebase. Nucleic acids research 42, D472–D477 (2014). 24243840 10.1093/nar/gkt1102 PMC3965010

[b5] Subramanian A. . Gene set enrichment analysis: a knowledge-based approach for interpreting genome-wide expression profiles. Proceedings of the National Academy of Sciences of the United States of America 102, 15545–15550, doi: 10.1073/pnas.0506580102 (2005). 16199517 PMC1239896

[b6] Huang da W., Sherman B. T. & Lempicki R. A. Systematic and integrative analysis of large gene lists using DAVID bioinformatics resources. Nature protocols 4, 44–57, doi: 10.1038/nprot.2008.211 (2009). 19131956

[b7] Maere S., Heymans K. & Kuiper M. BiNGO: a Cytoscape plugin to assess overrepresentation of gene ontology categories in biological networks. Bioinformatics 21, 3448–3449, doi: 10.1093/bioinformatics/bti551 (2005). 15972284

[b8] Zheng Q. & Wang X. J. GOEAST: a web-based software toolkit for Gene Ontology enrichment analysis. Nucleic Acids Res 36, W358–363, doi: 10.1093/nar/gkn276 (2008). 18487275 PMC2447756

[b9] Warde-Farley D. . The GeneMANIA prediction server: biological network integration for gene prioritization and predicting gene function. Nucleic acids research 38, W214–W220 (2010). 20576703 10.1093/nar/gkq537 PMC2896186

[b10] Zuberi K. . GeneMANIA prediction server 2013 update. Nucleic acids research 41, W115-W122 (2013). 23794635 10.1093/nar/gkt533 PMC3692113

[b11] Draghici S. . A systems biology approach for pathway level analysis. Genome Res. 17, 1537–1545 (2007). 17785539 10.1101/gr.6202607 PMC1987343

[b12] Voichita C. & Draghici S. ROntoTools: The R Onto-Tools suite. R package version 1 (2013).

[b13] Tarca A. L., Kathri P. & Draghici S. SPIA: Signaling Pathway Impact Analysis (SPIA) using combined evidence of pathway over-representation and unusual signaling perturbations. *R project Available:* http://bioinformatics.oxfordjournals.org/cgi/reprint/btn577v1 (2011).

[b14] Gu Z. & Wang J. CePa: an R package for finding significant pathways weighted by multiple network centralities. Bioinformatics , btt008 (2013). 10.1093/bioinformatics/btt008 23314125

[b15] Mitrea C. . Methods and approaches in the topology-based analysis of biological pathways. Frontiers in physiology 4 (2013). 10.3389/fphys.2013.00278 PMC3794382 24133454

[b16] Wang J. . NOA: a novel Network Ontology Analysis method. Nucleic Acids Res 39, e87, doi: 10.1093/nar/gkr251 (2011). 21543451 PMC3141273

[b17] Alexeyenko A. . Network enrichment analysis: extension of gene-set enrichment analysis to gene networks. BMC bioinformatics 13, 226 (2012). 22966941 10.1186/1471-2105-13-226 PMC3505158

[b18] Glaab E., Baudot A., Krasnogor N., Schneider R. & Valencia A. EnrichNet: network-based gene set enrichment analysis. Bioinformatics 28, i451–i457, doi: 10.1093/bioinformatics/bts389 (2012). 22962466 PMC3436816

[b19] Fang Z., Tian W. & Ji H. A network-based gene-weighting approach for pathway analysis. Cell research 22, 565–580 (2011). 21894192 10.1038/cr.2011.149 PMC3292304

[b20] Tomfohr J., Lu J. & Kepler T. B. Pathway level analysis of gene expression using singular value decomposition. BMC Bioinformatics 6, 225, doi: 10.1186/1471-2105-6-225 (2005). 16156896 PMC1261155

[b21] Tarca A. L., Carey V. J., Chen X. W., Romero R. & Draghici S. Machine learning and its applications to biology. PLoS Comput Biol 3 (2007). 10.1371/journal.pcbi.0030116 PMC1904382 17604446

[b22] Goeman J. J., van de Geer S. A., de Kort F. & van Houwelingen H. C. A global test for groups of genes: testing association with a clinical outcome. Bioinformatics 20, 93–99, doi: 10.1093/bioinformatics/btg382 (2003). 14693814

[b23] Tarca A. L., Draghici S., Bhatti G. & Romero R. Down-weighting overlapping genes improves gene set analysis. BMC Bioinformatics 13 (2012). 10.1186/1471-2105-13-136 PMC3443069 22713124

[b24] Michaud J. . Integrative analysis of RUNX1 downstream pathways and target genes. BMC genomics 9, 363, doi: 10.1186/1471-2164-9-363 (2008). 18671852 PMC2529319

[b25] Efron B. & Tibshirani R. On testing the significance of sets of genes. The Annals of Applied Statistics 1, 107–129, doi: 10.1214/07-aoas101 (2007).

[b26] Tarca A. L., Bhatti G. & Romero R. A comparison of gene set analysis methods in terms of sensitivity, prioritization and specificity. PloS one 8, e79217, doi: 10.1371/journal.pone.0079217 (2013). 24260172 PMC3829842

[b27] Latora V. & Marchiori M. Efficient behavior of small-world networks. Physical review letters 87, 198701 (2001). 11690461 10.1103/PhysRevLett.87.198701

[b28] Knijnenburg T. A., Wessels L. F., Reinders M. J. & Shmulevich I. Fewer permutations, more accurate P-values. Bioinformatics 25, i161–i168 (2009). 19477983 10.1093/bioinformatics/btp211 PMC2687965

[b29] Hochberg Y. A sharper Bonferroni procedure for multiple tests of significance. Biometrika 75, 800–802 (1988).

[b30] Narum S. R. Beyond Bonferroni: Less conservative analyses for conservation genetics. Conservation Genetics 7, 783–787, doi: 10.1007/s10592-005-9056-y (2006).

[b31] Alexeyenko A. . Comparative interactomics with Funcoup 2.0. Nucleic Acids Res 40, D821–828, doi: 10.1093/nar/gkr1062 (2012). 22110034 PMC3245127

[b32] Hao L. . Limited Agreement of Independent RNAi Screens for Virus-Required Host Genes Owes More to False-Negative than False-Positive Factors. Plos Computational Biology 9, doi: 10.1371/journal.pcbi.1003235 (2013). PMC3777922 24068911

[b33] Karlas A. . Genome-wide RNAi screen identifies human host factors crucial for influenza virus replication. Nature 463, 818–822 (2010). 20081832 10.1038/nature08760

[b34] Hao L. . Drosophila RNAi screen identifies host genes important for influenza virus replication. Nature 454, 890–893 (2008). 18615016 10.1038/nature07151 PMC2574945

[b35] Brass A. L. . The IFITM proteins mediate cellular resistance to influenza A H1N1 virus, West Nile virus, and dengue virus. Cell 139, 1243–1254 (2009). 20064371 10.1016/j.cell.2009.12.017 PMC2824905

[b36] König R. . Human host factors required for influenza virus replication. Nature 463, 813–817 (2009). 20027183 10.1038/nature08699 PMC2862546

[b37] Ashburner M. . Gene Ontology: tool for the unification of biology. Nature genetics 25, 25–29 (2000). 10802651 10.1038/75556 PMC3037419

[b38] Csardi G. & Nepusz T. The igraph software package for complex network research. InterJournal, Complex Systems 1695 (2006).

[b39] Kim H. . YeastNet v3: a public database of data-specific and integrated functional gene networks for Saccharomyces cerevisiae. Nucleic acids research 42, D731–D736 (2014). 24165882 10.1093/nar/gkt981 PMC3965021

[b40] Szklarczyk D. . The STRING database in 2011: functional interaction networks of proteins, globally integrated and scored. Nucleic acids research 39, D561–D568 (2011). 21045058 10.1093/nar/gkq973 PMC3013807

[b41] Franceschini A. . STRING v9. 1: protein-protein interaction networks, with increased coverage and integration. Nucleic acids research 41, D808–D815 (2013). 23203871 10.1093/nar/gks1094 PMC3531103

[b42] Lee I., Blom U. M., Wang P. I., Shim J. E. & Marcotte E. M. Prioritizing candidate disease genes by network-based boosting of genome-wide association data. Genome research 21, 1109–1121 (2011). 21536720 10.1101/gr.118992.110 PMC3129253

[b43] Smyth G. K. Linear models and empirical Bayes methods for assessing dierential expression in microarray experiments. Statistical Applications in Genetics and Molecular Biology 3, 3 (2004). 10.2202/1544-6115.1027 16646809

[b44] Smyth G. K. Limma: linear models for microarray data. , 397–420 (Springer, 2005).

[b45] Khatri P. . Recent additions and improvements to the Onto-Tools. Nucleic Acids Res 33, 762–765 (2005). 10.1093/nar/gki472 PMC1160233 15980579

[b46] Khatri P. . Onto-Tools: new additions and improvements in 2006. Nucleic Acids Res. 37, 206–211 (2007). 10.1093/nar/gkm327 PMC1933142 17584796

[b47] Voichita C., Donato M. & Draghici S. in Machine Learning and Applications (ICMLA) 2012 11th International Conference Vol. 1 126–131 (IEEE, Boca Raton, FL, 2012).

[b48] Tarca A. L. . A novel signaling pathway impact analysis (SPIA). Bioinformatics 25, 75–82 (2009). 18990722 10.1093/bioinformatics/btn577 PMC2732297

[b49] Gu Z., Liu J., Cao K., Zhang J. & Wang J. Centrality-based pathway enrichment: a systematic approach for finding signif- icant pathways dominated by key genes. BMC Syst. Biol. 6 (2012). 10.1186/1752-0509-6-56 PMC3443660 22672776

[b50] Voichita C., Donato M. & Draghici S. Incorporating gene significance in the impact analysis of signaling pathways. *Proceedings of the International Conference on Machine Learning Applications (ICMLA)* (2012).

[b51] Bradley Efron R. T. On testing the significance of sets of genes. Annals of Applied Statistics 1, 107–129 (2006).

[b52] Sun S., Dong X., Fu Y. & Tian W. An iterative network partition algorithm for accurate identification of dense network modules. Nucleic Acids Research 40, doi: 10.1093/nar/gkr1103 (2012). PMC3273790 22121225

[b53] De Rubeis S. . Synaptic, transcriptional and chromatin genes disrupted in autism. Nature 515, 209–215, doi: 10.1038/nature13772 (2014). 25363760 PMC4402723

[b54] Iossifov I., O’Roak B. J. & Sanders S. J. The contribution of de novo coding mutations to autism spectrum disorder. Nature (2014). 10.1038/nature13908 PMC4313871 25363768

[b55] Luscombe N. M. . Genomic analysis of regulatory network dynamics reveals large topological changes. Nature 431, 308–312 (2004). 15372033 10.1038/nature02782

[b56] Malumbres M. & Barbacid M. To cycle or not to cycle: a critical decision in cancer. Nature Rev. Cancer 1, 222–231 (2001). 11902577 10.1038/35106065

[b57] Braakman I., Hoover-Litty H., Wagner K. R. & Helenius A. Folding of influenza hemagglutinin in the endoplasmic reticulum. The Journal of cell biology 114, 401–411 (1991). 1650370 10.1083/jcb.114.3.401 PMC2289100

[b58] Rual J. F. . Towards a proteome-scale map of the human protein-protein interaction network. Nature 437, 1173–1178, doi: 10.1038/nature04209 (2005). 16189514

[b59] Kocovska E., Fernell E., Billstedt E., Minnis H. & Gillberg C. Vitamin D and autism: Clinical review. Research in Developmental Disabilities 33, 1541–1550, doi: 10.1016/j.ridd.2012.02.015 (2012). 22522213

[b60] Hussman J. P. Suppressed GABAergic inhibition as a common factor in suspected etiologies of autism. Journal of Autism and Developmental Disorders 31, 247–248, doi: 10.1023/a:1010715619091 (2001). 11450824

[b61] Su S. & Mitchell A. P. Identification of functionally related genes that stimulate early meiotic gene expression in yeast. Genetics 133, 67–77 (1993). 8417990 10.1093/genetics/133.1.67 PMC1205299

[b62] Karpichev I. V. & Small G. M. Global regulatory functions of Oaf1p and Pip2p (Oaf2p), transcription factors that regulate genes encoding peroxisomal proteins in Saccharomyces cerevisiae. Molecular and cellular biology 18, 6560–6570 (1998). 9774671 10.1128/mcb.18.11.6560 PMC109241

[b63] Harris S. L. & Levine A. J. The p53 pathway: positive and negative feedback loops. Oncogene 24, 2899–2908, doi: 10.1038/sj.onc.1208615 (2005). 15838523

[b64] Hammond E. M. & Giaccia A. J. The role of p53 in hypoxia-induced apoptosis. Biochemical and biophysical research communications 331, 718–725, doi: 10.1016/j.bbrc.2005.03.154 (2005). 15865928

[b65] Bauer S., Gagneur J. & Robinson P. N. GOing Bayesian: model-based gene set analysis of genome-scale data. Nucleic acids research 38, 3523–3532 (2010). 20172960 10.1093/nar/gkq045 PMC2887944

