# Supplementary material for: LEGO: a novel method for gene set over-representation analysis by incorporating network-based gene weights
Source: Sci Rep. 2016 Jan 11;6:18871. doi: 10.1038/srep18871 (PMC4707541; doi:10.1038/srep18871)
Supplement: Supplementary Information [file srep18871-s1.doc]

LEGO: a novel method for gene set over-representation analysis by incorporating network-based gene weights

Xinran Dong^1,2^, Yun Hao^1,2^, Xiao Wang^1^, and Weidong Tian^1,*^

Authors’ affiliations:

^1^State Key Laboratory of Genetic Engineering, Department of Biostatistics and Computational Biology, School of Life Sciences, Fudan University, Shanghai 200436, P.R. China

Author Notes:

^2^: The authors wish it to be known that, in their opinion, the first two authors should be regarded as joint First Authors.

^*^: Corresponding Author: Weidong Tian, State Key Laboratory of Genetic Engineering, Department of Biostatistics and Computational Biology, School of Life Sciences, Fudan University, Shanghai, 200436, P.R. China.

**Summary for the supplementary materials:**

**Supplementary Notes:**

Note1: Detailed process of p-value calculation.

**Supplementary Figures:**

Figure S1: Boxplots of the frequency of random significance of all 1,070 MSigDB gene sets using LEGO.

FigureS2: The impact of network features on the performance of different methods.

FigureS3: The impact of network perturbation on the performance of LEGO in the yeast datasets.

FigureS4: Spearman correlation of the gene rank calculated by LEGO based on FunCoup and STRING network.

FigureS5: The impact of network choice on the performance of LEGO.

**Supplementary Tables:**

TableS1: Summary for the results of yeast datasets.

TableS2: Summary for the results of human datasets.

TableS3: Comparison of calculation time for, LEGO, NEA, EnrichNet and Fisher in testing datasets.

TableS4: Results of LEGO and other 10 algorithms for 34 gene expression datasets

TableS5: Summary of the results for the autism datasets.

TableS6: Gene weight score for the selected genes in all MSigDB gene sets.

**Supplementary Note:**

**Note1: Detailed process for p-value estimation.**

Given observation score *X_0_* and *N* permutation scores *Y_1_…Y_N_*, the permutation p-value is given as:

$$P_{perm}=\frac{1+\sum_{n=1}^{N} I(Y_{n}\geq X_{0})}{N}$$

where I(.) is the indicator function. If the *P_perm_* is larger than the threshold α，we will report it as the final p-value. If not, we will use GPD (generalized Patero distribution) approximation to get the exact p-value.

Firstly, we sort all permutation score with the decreasing trend and extract out the most extreme 250 scores. Then, we treat the mean value of the 250^th^ and 251^th^ score as exceedance threshold *t*. Thus, we could get 250 exceedance score *Z_1_…Z_250_*, where *Z_n_=Y_n_-t.*

Then, we use good-of-fitness test to approximate the 250 *Z_n_* score to GPD. If p-value is smaller than 0.05, we will iteratively decrease the number of exceedance by ten until a good fit (p-value >0.05). When the number of exceedance is smaller than 100 but the p-value is still less than 0.05, we will use the permutation p-value instead.

Finally, if the exceedance score could fit GPD, we will adjust the permutation p-value by:

$$P_{gpd}=P_{perm}*(1-F(X_{0}-t))$$

where F(.) is the cumulative function of the GPD. The flow chart is summarized as follows:

Given:

*Observation score: X_0_;*

*N permutation score: Y_1_…Y_N_; Sorted with decreasing trend.*

Compute:

*M=length(which(Y_i_>=X_0_))+1;*

*T=(Y_N-251_+Y_N-250_)/2;*

*Z_i_= Y_i_-T;*

Test*:*

*Whether Z_i_ follow GPD by goodness-of-fit test.*

*If p_GPD_ < 0.05, iteratively decrease the number of Z_i_ by ten until a GPD good fit.*

Final p-value:

If *M/N >αor GPD fit failed, use*:

*p-value*: *M/N;*

else:

*permutation p-value: M/N;*

*adjusted p-value: M/N*(1-F(X_0_-T)); Where F is the cumulative distribution function of GPD*

**Supplementary Figures:**

**Supplementary Figure S1| Boxplots of the frequency of random significance of all 1,070 MSigDB gene sets using LEGO.** Each time, 200 genes were randomly selected and were subjected for LEGO analysis; the gene sets found significant (p-value < 0.05) were recorded. The frequency that a gene set was found significant (termed as frequency of random significance) was computed when the experiment was repeated 100, 1000, and 10000 times, and the corresponding boxplots were plotted, respectively.

**Supplementary Figure S2 | The impact of network features on the performance of different methods.** **(a)** All 1,077 MSigDB gene sets are divided into five categories based on their size. Each category contains boxplots of the frequency of random significance with 200 randomly selected genes in the human dataset. Similar to **(b),** all 1,077 gene sets are divided into five categories based on their odds ratio for the gene set network Efficiency (NE) values (the odds ratio is calculated by their original NE value divided by the NE value of the background network). In the box inside the two figures, the number after each algorithm name is the Pearson Correlation Coefficient value between the frequency of random significance and the gene set size (or the odds ratio for the gene set NE value).

**Supplementary Figure S3 | The impact of network perturbation on the performance of LEGO in the yeast datasets. (a,b)** Scatter plots for the average rank of the two relevant GO term (GO:0022402, GO:0043934) identified by LEGO at different portions of edge removal in the Yeast TF network. **(c,d)** Similar to **(a,b),** except that a selected portion of edges are randomly rewired.

**Supplementary Figure S4| Spearman correlation of the gene rank calculated by LEGO based on FunCoup and STRING network.** For each of 1,077 MSigDB gene sets, we calculate the spearman correlation between the gene ranks (based on gene weight) based on the STRING network and those based on the FunCoup network. Then, the distribution of correlation for all 1,077 gene sets is plotted.

**Supplementary Figure S5 | The impact of network choice on the performance of LEGO.** For network choice, **(a)** shows the number of overlapped significant MSigDB gene sets (out of top 80) in the human dataset identified by LEGO using different background networks (FunCoup, STRING and HumanNet), **(b)** shows the Jaccard similarity between the overlapped pathways (top 80) identified by LEGO using different networks, which is defined as the intersection / the number of the smaller list.

**Supplementary Tables:**

**Supplementary Table S3 | Comparison of calculation time for, LEGO, NEA, EnrichNet and Fisher in testing datasets.**

| **Dataset** | **LEGO** | **NEA** | **EnrichNet** | **Fisher** |
| --- | --- | --- | --- | --- |
| **The yeast TF dataset** | <1s | 237s | < 1s | < 1s |
| **The three breast cancer datasets** | 161s | 179955s | 138s | 6s |
| **The four influenza datasets** | 900s | 170 hr | 300s | 10s |
